# Shortening the adaptation of Nellore cattle to high-concentrate diets using only virginiamycin as sole feed additive negatively impacts ruminal fermentation and nutrient utilization

**DOI:** 10.3389/fvets.2023.1089903

**Published:** 2023-03-29

**Authors:** Mariana M. Squizatti, André L. N. Rigueiro, Antonio M. Silvestre, Carlos H. G. Soares, Alice H. P. M. Assumpção, Evandro F. F. Dias, Luana D. Felizari, Leandro A. F. Silva, Katia L. R. Souza, Victor M. Carvalho, Breno L. Demartini, Johnny M. Souza, Danilo D. Millen

**Affiliations:** ^1^School of Veterinary Medicine and Animal Science, São Paulo State University (UNESP), Botucatu, São Paulo, Brazil; ^2^College of Agricultural and Technological Sciences, São Paulo State University (UNESP), Dracena, São Paulo, Brazil

**Keywords:** rumen, metabolism, feedlot, protozoa, degradability

## Abstract

Feedlot cattle are usually adapted to high-concentrate diets containing sodium monensin (MON) in more than 14 days. However, considering that the dry matter intake DMI is usually lower during adaptation when compared to the finishing period, the use of MON during adaptation may decrease even further the DMI, and virginiamycin (VM) may be an alternative. This study was designed to investigate the effects of shortening the adaptation length from 14 to 9 or 6 days on ruminal metabolism, feeding behavior, and nutrient digestibility of Nellore cattle fed high-concentrate diets containing only VM as the sole feed additive. The experimental design was a 5 × 5 Latin square, where each period lasted 21 days. Five 17 mo-old Nellore yearling bulls were used (415 ± 22 kg of body weight), which were assigned to five treatments: (1) MON (30 mg/kg) and adaptation for 14 days; (2) MON (30 mg/kg) + VM (25 mg/kg) and adaptation for 14 days; (3) VM (25 mg/kg) and adaptation for 14 days; (4) VM (25 mg/kg) and adaptation for 9 days, and (5) VM (25 mg/kg) and adaptation for 6 days. A quadratic effect for adaptation length when only VM was fed was observed for mean pH (*P* = 0.03), duration of pH below 5.2 (*P* = 0.01) and 6.2 (*P* = 0.01), where cattle consuming VM adapted for 9 days had higher mean pH and shorter period of pH below 5.2 and 6.2. Cattle that consumed only MON had a lower concentration of butyrate (*P* = 0.02) and a higher concentration of propionate (*P* = 0.04) when compared to those consuming VM and adapted for 14 days. As the adaptation length decreased for animals consuming only VM, the rumen degradability of dry matter (*P* < 0.01), neutral detergent fiber (*P* < 0.01), and starch (*P* < 0.01) decreased; however, protozoa numbers of *Entodinium* and total protozoa increased. It is not recommended to shorten the adaptation length of these animals to either 6 or 9 days without negatively impacting nutrient disappearance and ruminal fermentation patterns.

## 1. Introduction

Ruminant animals, including beef cattle, evolved as herbivores, consuming diets where forages were predominant. However, to supply the increasing demand worldwide for beef, the adoption of feedlot systems became a reality, and grains and concentrate feedstuffs became most of the diets consumed by beef cattle in these systems (1, 2). Furthermore, high-concentrate or starch-rich diets modify the ruminal microflora (3), and this transition from a forage-based to a high-grain diet should take place gradually to allow microorganisms, as well as the ruminal epithelium, to adapt properly to the increasing amount of readily fermentable carbohydrates in the rumen (4).

Several studies have been conducted in North America (5, 6) and Brazil (7–11) to investigate the most appropriate adaptation length to high-concentrate diets for feedlot cattle. Brown et al. (5) compiled several studies from North America and reported that feedlot cattle should not be adapted in < 14 d to high-concentrate diets without impairing overall performance and health. Concerning Brazilian feedlots, based on the results of the studies cited above, it was found that, regardless of the type of protocol used, corn processing method, and nutritional background, the adaptation period for Nellore cattle in Brazil should not be in < 14 d as well.

For feedlot systems, typically, sodium monensin (MON) is included in the diets in North America and Brazil (1, 2) to improve feed efficiency (12) by reducing dry matter intake (DMI), and to minimize ruminal acidification. Furthermore, all adaptation studies from Brazil mentioned earlier fed high-concentrate diets containing MON as the sole feed additive.

Therefore, it is well documented in the literature that the DMI is usually lower during adaptation when compared to the finishing period (13) and the use of a feed additive, such as the MON, may decrease even further the DMI in this period. Since finishing diets in Brazil contain less energy (1.22 vs. 1.52 Mcal/kg of diet dry matter) than a typical feedlot diet in North America (1, 2), the use of feed additives that decrease DMI, especially during adaptation, may not be recommended. In this context, Virginiamycin (VM) is a growth promoter, and a potential MON replacement in this scenario, that may improve feedlot performance without negatively impacting DMI (14), and it was the second feed additive mostly used by Brazilian nutritionists in 2019 (2). In a Brazilian study, authors reported that Nellore bulls fed VM, as the sole feed additive during adaptation, reached DMI of 2% of body weight (BW) in 4.3 d, on average; whereas that fed MON needed 20.7 d to reach a similar intake (13). In this context, the faster cattle reach a DMI of 2% of BW, the more adapted they are to the diets, since DMI is an important indicator to evaluate how well cattle are either accepting or adapted to the diets (5). As a result, the use of VM as the sole feed additive in feedlot diets containing a moderate amount of energy may allow the adaptation period to be shortened to nine or even 6 days, which may represent a greater economic return, since the animals will be adapted to the finishing diet earlier. Thus, this study was designed to investigate the effects of shortening the adaptation length from 14 to 9 or 6 days on ruminal metabolism, feeding behavior, and nutrient digestibility of Nellore cattle fed high-concentrate diets containing only VM as the sole feed additive.

## 2. Materials and methods

All the procedures involving the use of animals in this study were in accordance with the guidelines established by the São Paulo State University Ethical Committee for Animal Research (protocol number 02/2017.R1- CEUA).

### 2.1. Animals and treatments

The trial was conducted at the São Paulo State University feedlot, Dracena campus, Brazil. Five 22-mo-old yearling Nellore steers (414.86 ± 21.71 kg) fitted with ruminal cannulas were randomly assigned to a 5 × 5 Latin square design. Cattle were randomly assigned to a different treatment in each period, which lasted 21 days. Therefore, the experimental treatments were as follows: (1) MON [27 mg/kg of dry matter (DM)] and 14-d adaptation (MON14); (2) MON (27 mg/kg of DM) + VM (25 mg/kg of DM) and 14-d adaptation (MONVM14); (3) VM (25 mg/kg of DM) and 14-d adaptation (VM14); (4) VM (25 mg/kg of DM) and 9-d adaptation (VM9); e (5) VM (25 mg/kg of DM) and 6-d adaptation (VM6).

### 2.2. Feeding and management description

At the beginning of the study, all steers were dewormed and vaccinated (tetanus, bovine viral diarrhea virus, 7-way *Clostridium sp*.; Cattlemaster and Bovishield, Pfizer Animal Health, New York, NY). Nellore steers were housed in individual pens (72 m^2^) equipped with 6 m of linear bunk space and free water access to a drinking fountain (3.00 × 0.80 × 0.20 m) shared by two animals. Steers were fed *ad libitum* with a total mixed ration (TMR) once a day at 0800 h, and DMI was calculated daily by weighing ration offered and orts, before the next morning delivery, and expressed both in kilograms and as a percentage of BW. The dietary DM was determined daily following the procedures from (method 934.01; AOAC, 1990). The amount of feed offered was adjusted daily based on the targeted amount of orts (3 to 5%) left before morning feed delivery (0700 h). The BW was measured at the beginning (day 1) and at the end (day 21) of each period at 0700 h.

The basal diets were formulated according to the Large Ruminant Nutrition System (15) and are shown in Table 1. Basal diets were composed of sugarcane bagasse, *Cynodon dactylon hay*, corn grain (finely ground), soybean meal, mineral supplement, and urea. The step-up adaptation program consisted of *ad libitum* intake with increasing levels of concentrate ingredients until reaching the concentrate level of the finishing diet (84%). Adaptation diets 1, 2, and 3 contained 66, 72, and 78% concentrate. Moreover, the management of the adaptation diets was performed according to treatments as follows: cattle adapted for 6 d were fed adaptation diets for two d each; animals adapted for 9 d received adaptation diets for three d each; whereas cattle adapted for 14 d were fed 66, 72, and 78% concentrate for 5 d, 4 d, and 5 d, respectively.

**Table 1:** Feed ingredients and chemical composition of high-concentrate diets fed to Nellore yearling bulls during adaptation and finishing periods.

**Item**	**Percent of concentrate**			
	**66**	**72**	**78**	**84**
**Ingredients, % of DM** ^a^				
Sugarcane bagasse	20.00	18.00	16.00	12.00
*Cynodon dactylon* hay	15.00	10.00	5.00	2.00
Finely ground corn grain	41.80	50.00	59.60	70.00
Soybean meal	20.00	18.70	16.00	12.55
Supplement^b^	2.50	2.50	2.50	2.50
Urea	0.70	0.80	0.90	0.95
**Nutrient content, % of DM** *^c^*				
DM, as % of organic matter	46.00	48.00	51.00	57.00
Total digestible nutrients	64.00	67.00	70.00	74.00
Crude protein	15.60	15.60	15.20	14.60
Neutral detergent fiber	41.40	36.60	31.40	14.60
Non-fiber carbohydrates	38.00	43.00	49.00	55.00
peNDF^d^	28.00	23.00	18.00	13.00
NEg^e^, Mcal/kg^e^	1.00	1.08	1.15	1.26
Ca	0.60	0.58	0.56	0.54
P	0.40	0.41	0.42	0.42

^a^Dry matter;

^b^Supplement contained: Ca: 182g/kg of DM; P: 40.5g/kg of DM; Mg: 7.7g/kg of DM; K: 0.5g/kg of DM; Na: 82.2g/kg of DM; Cl: 126.5g/kg of DM; S: 16g/kg of DM; Co: 27.50 mg/kg of DM; Cu: 754.17 mg/kg of DM; Fe: 2498 mg/kg of DM; I: 37.29 mg/kg of DM; Mn: 740 mg/kg of DM; Se: 6.20 mg/kg of DM; Zn: 1790 mg/kg of DM. Monensin (Bovensin 200; Phibro Animal Health Corporation, Guarulhos, São Paulo, Brazil) was added at 1000 mg/kg of supplement and Virginiamycin (V-Max 2; Phibro Animal Health Corporation, Guarulhos, São Paulo, Brazil) was added at 833 mg/kg of supplement and offered to yearling bulls according to the treatments;

^c^Estimated by equations according to Large Ruminant Nutrition System Fox et al., (15);

^d^Phisically effective NDF determined according to method described by Heinrichs and Kononoff (18);

^e^Net energy for gain, Mcal.

### 2.3. Experimental period

**Figure 1:**
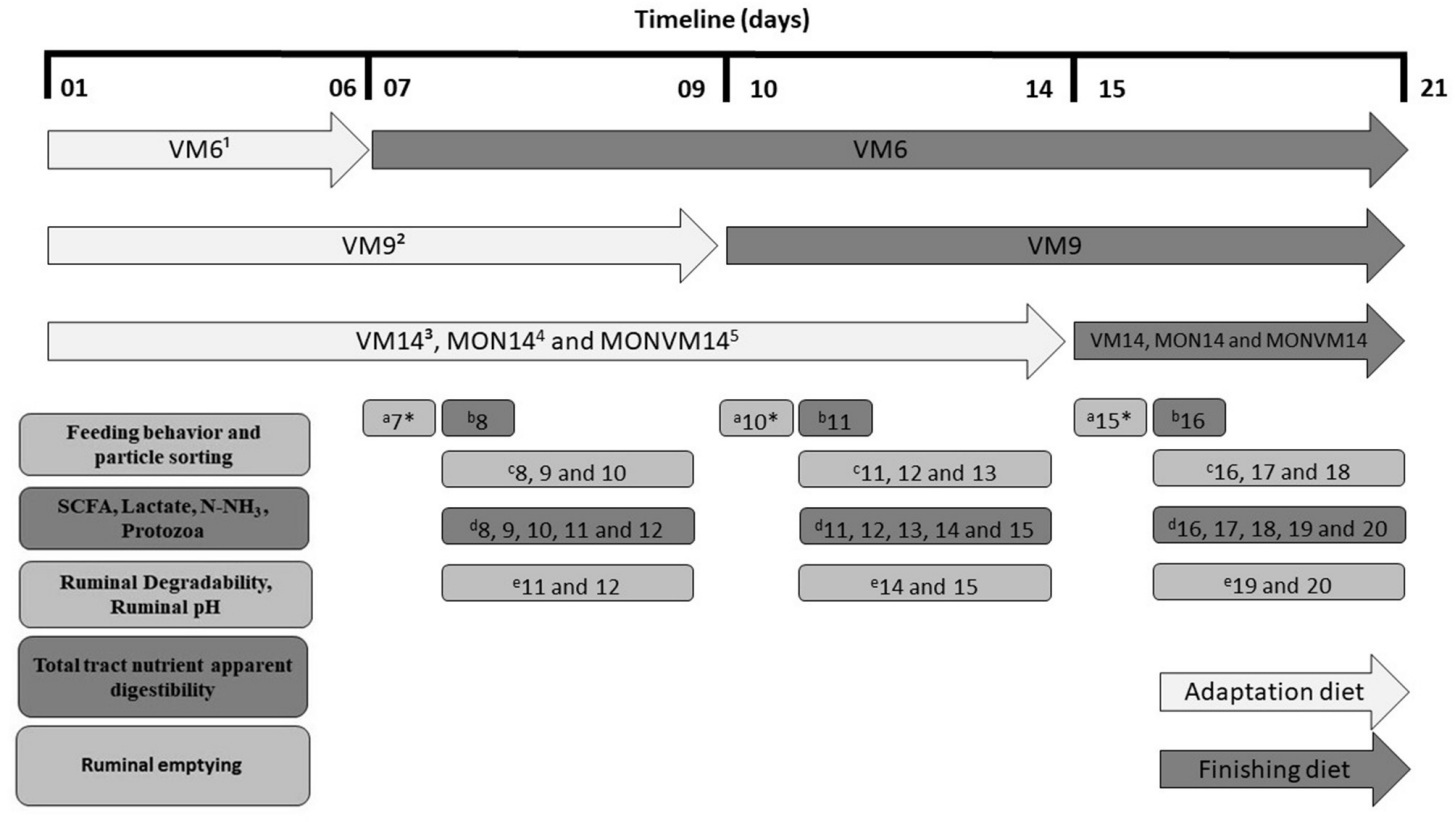
Timeline of samples and data collection from Nellore steers cannulated in the rumen and fed Virginiamycin as sole feed additive in each experimental period. ^1^VM6: 25 mg of VM/kg of DM and adaption for 6 d; ^2^VM9: 25 mg of VM/kg of DM and adaption for 9 d; ^3^VM14: 25 mg of VM/kg of DM and adaption for 14 d; ^4^MON14: 27 mg of MON/kg of DM and adaption for 14 d; ^5^MON+VM14: 27 mg of MON/kg of DM and 25 mg of VM/kg of DM and adaption for 14 d; ^a^Feeding behavior and particle sorting; ^b^Short chain fatty acids, lactate, N-NH_3_ and Protozoa; ^c^Ruminal degradability and Ruminal pH; ^d^Total tract nutrient apparent digestibility; ^e^Ruminal emptying. ^*^Sampling was performed at days 7 to 12 for animals fed VM6 only (6-d adaptation); days 10 to 15 for animals fed VM9 only (9-d adaptation); days 15 to 16 for all animals (VM14, MON14, and MONVM14).

All data and samples in this study were collected, from each experimental period, according to the timeline shown in Figure 1. The description of each method employed to process and analyze samples and data collected are described in the following sections.

### 2.4. Feeding behavior and particle sorting

Cattle were submitted to visual observations to evaluate feeding behavior, every 5 min, over 24 h on day 07 (only animals fed VM6; 6-d adaptation), day 10 (only animals fed VM9; 9-d adaptation), and day 15 (all animals) of each experimental period 15 of each experimental period. The visual observations were performed according to Robles et al. (16). Feeding behavior data were recorded for each animal as follows: time spent eating, ruminating, and resting (expressed in minutes), and the number of meals per day. A meal was considered the non-interrupted time cattle stayed in the feed bunk eating the ration.

The meal length in minutes was calculated by dividing the time spent eating by the number of meals per day. The DMI per meal in kilograms was calculated by dividing DMI by the number of meals per day. In addition, time spent eating and time spent ruminating data were used to calculate the eating rate of DM (ERDM; time spent eating/DMI) and rumination rate of DM (RRDM; time spent ruminating/DMI), both expressed in minutes per kilogram of DM. Moreover, samples of diets and orts were collected on the days of feeding behavior data collection for chemical analysis of neutral detergent fiber (NDF) (17) to determine the intake of NDF. The eating rate of NDF (ERNDF) was then calculated by dividing the time spent eating by NDF intake. Likewise, the rumination rate of NDF (RRNDF) was determined by dividing the time spent ruminating by NDF intake. Both ERNDF and RRNDF were expressed in minutes per kilogram of NDF.

Samples of diets and orts were also collected for determination of particle-size distribution, which was performed by sieving using the Penn State Particle Size Separator and reported on an as-fed basis as described by Heinrichs and Kononoff (18). Particle sorting was determined as follows: n intake/n predicted intake, in which *n* = particle fraction screens of 19 mm (long), 8 mm (medium), 1.18 mm (short), and a pan (fine). Particle sorting values equal to 1 indicate no sorting, those < 1 indicate selective refusals (sorting against), and those >1 indicate preferential consumption [sorting for; (19)].

### 2.5. *In situ* degradability

The *in situ* degradability determination was performed on days 8, 9 and 10 (only animals fed VM6; 6-d adaptation); days 11, 12 and 13 (only animals fed VM9; 9-d adaptation); and days 16, 17 and 18 (all animals) of each experimental period, according to methodology adapted from Mehres and Ørskov (20). About 15 g of diet samples, previously dried at 65°C for 72 h, were added to nylon bags with a porosity of 50 microns, measuring 10.0 × 19.0 cm. Bags were inserted into the rumen and incubated for 72 h. After rumen retrieval, nylon bags were washed with cold running water, and then oven-dried at 65°C for 72 h. Samples not incubated in the rumen were also washed as described above. Samples were analyzed for DM [method 934.01; (21)]; crude protein (CP), by total N determination using the micro-Kjeldahl technique[method 920.87; (21)], NDF, with heat-stable α-amylase according to Van Soest et al. (17), and acid detergent fiber (ADF) according to Van Soest et al. (17). Starch analysis was performed according to Pereira and Rossi (22), with the previous extraction of soluble carbohydrates, as proposed by Hendrix (23). Ether extract (EE), was determined gravimetrically after extraction using petroleum ether in a Soxhlet extractor [method 920.85 (21)]. The value of nitrogen-free extract (NFE) and total digestible nutrients (TDN) was estimated according to NRC (24). The apparent coefficient of nutrient degradability was calculated by the following equation: 100 × [01 – (bag weight after incubation – empty bag weight) / (bag weight before incubation – empty bag weight)].

### 2.6. Ruminal fermentation variables

Ruminal pH was continuously measured every 10 min, on days 8, 9 and 10 (only animals fed VM6; 6-d adaptation); days 11, 12 and 13 (only animals fed VM9; 9-d adaptation); and days 16, 17 and 18 (all animals) of each experimental period, using a Lethbridge Research Center Ruminal pH Measurement System (LRCpH; Dascor, Escondido, CA) as described by Penner et al. (25). The pH electrode (model T7-1 LRCpH, Dascor, Escondido, CA model S650) was covered by a shroud that allowed particle and liquid passage but kept the pH electrode from contacting the surface of the ruminal epithelium. The capsule was attached to the ruminal cannula plug to aid in system location within the rumen and to help maintain the electrode in a vertical position. Two 900-g weights were fastened to the bottom of the electrode shroud to maintain the electrode in the ventral sac of the rumen. Readings in pH buffers 4 and 7 were recorded before placing the LRCpH system in the rumen. The daily ruminal pH data were averaged and summarized as minimum pH, mean pH, and maximum pH, as well as the area under the curve, and the duration of time in which pH was below 6.2, 6.0, and 5.8. The area under the curve was calculated by multiplying the absolute value of deviations in pH by the time (min) spent below the established threshold for each measure divided by 60 and expressed as pH unit × hour. Likewise, data loggers recorded rumen temperature and ox-redox potential (25).

Ruminal fluid samples were collected *via* cannula at 0, 3, 6, 9, and 12 h after the morning meal on days 8, 9 and 10 (only animals fed VM6; 6-d adaptation); days 11, 12 and 13 (only animals fed VM9; 9-d adaptation); and days 16, 17 and 18 (all animals) of each experimental period. Approximately 500 mL of rumen fluid was collected, at each sampling time, from 3 different parts of the rumen. After the collection of samples, the remaining ruminal fluid was returned to the rumen immediately after the collection. For short-chain fatty acid (SCFA) analyses that included acetate, propionate, and butyrate, a fraction of approximately 100 mL of ruminal fluid was centrifuged at 2,000 × g for 20 min at room temperature, and 2 mL of the supernatant was added to 0.4 mL of formic acid and frozen at −20°C for further analyses, according to Erwin et al. (26). The SCFA were measured by gas chromatography (Finnigan 9001, Thermo Scientific, West Palm Beach, FL) using a glass column Ohio Valley Megabore, model 1 OV-351 of 1 Micron, being 30 mm long and 0.53 mm in diameter. Lactic acid concentration was measured by a colorimetric technique, according to Erwin et al. (26). For NH_3_-N concentration determination, 2 mL of the supernatant was added to 1 mL of 1 N of H_2_SO_4_ solution and the centrifuge tubes were immediately frozen until the colorimetric analyses, according method described by Kulasek (27) and adapted by Foldager (28).

### 2.7. Ruminal protozoa counting

For the differential counting of rumen ciliated protozoa, the ruminal content was manually collected by sweeping the floor of this organ, and 10 ml of this material was stored in a vial containing 20 ml of 50% (v / v) formaldehyde. The collections were performed on day 10 (only animals fed VM6; 6-d adaptation); day 13 (only animals fed VM9; 9-d adaptation); and day 18 (all animals) of each experimental period, at 0, 4, 8, and 12 hours after morning feeding. In 1-mL sample was added two drops of 2% brilliant green and diluted with 9 ml of 30% glycerol. Protozoa were identified (genus *Isotricha, Dasytricha, Entodinium* and *Diplodiniinae* subfamily) and counted using a Sedgwick counting chamber Rafter with internal dimensions of 50 mm × 20 mm × 1 mm (capacity 1 mL) by optical microscopy (Olympus CH-2^®^, Japan) (29).

### 2.8. Total tract apparent digestibility

The apparent total tract digestibility was determined using titanium dioxide (TiO_2_) as an external marker according to Pezzato et al. (30). Cattle received 12 g of titanium dioxide daily, through the ruminal cannula, from day 11 to day 20 of each experimental period. Diet and orts samples were collected from each pen from day 16 to 20 once a day at 0800 h. Samples of feces were collected from day 16 to 20 of each experimental period twice a day at 0800 h and at 1600 h. Feed and fecal samples were dried at 65°C for 72 h and ground to pass a 1-mm screen. After being individually ground, each daily sample was appropriately weighed and an aliquot of each day was taken to compose an individual composite sample per animal per period (~200 g). Composite samples per animal were used to determine DM [method 934.01; (21)]; CP [method 920.87; (21)]; NDF and ADF (17); starch (22) as proposed by Hendrix (23); EE [method 920.85; (21)]; NFE and total TDN (24), as described above for *in situ* degradability of nutrients. Titanium dioxide concentration was determined according to Pezzato et al. (30). The digestibility coefficients were calculated based on the titanium dioxide (TiO_2_) content of feces samples. The excretion of DM and nutrients, as well as nitrogen excretion, were calculated from the digestibility coefficient data of DM and their fractions, multiplying the nutrient intake by the respective digestibility coefficients and dividing by 100.

### 2.9. Ruminal dynamics

The ruminal digesta was removed manually from each steer through the rumen cannula to determine the disappearance rate in the rumen as described by Dado and Allen (31). On day 11 (VM6); day 14 (VM9); and day 19 (all treatments) of each experimental period, steers had their rumens emptied at 11:00, which was about 3 h after delivering the morning meal, based on assumption that the rumen is at the highest level of volume. The same procedure was done on day 12 (VM6); day 15 (VM9); and day 20 (all treatments) of each experimental period, at 8:00, immediately before the morning meal delivery, assuming that the rumen is at its lowest volume. During the emptying procedure of ruminal contents, liquid and solid phases were separated, weighed, and then a 1 kg sample from each steer was homogenized taking into account the proportion of liquid and solid phases for determination of DM. Consequently, rumen digesta was reconstituted and placed back in the rumen of the steer it originally came from. The rumen pool of DM and its disappearance rate were calculated based on the dry weight of each sample (55°C for 72 h). The DM disappearance rate was considered equal to the intake rate, and they were estimated using the formula (32): DM disappearance rate (%/h) = Daily DM intake (kg) / DM Ruminal contents (kg) /24.

### 2.10. Statistical analysis

First, data related to days 15 to 20 were analyzed, in which all treatments were in the finishing phase, after 14 days of adaptation (minimum period to adapt cattle to finishing diets). Subsequently, data were analyzed shortly after the start of finishing phase, which would be days 7 to 11 and 10 to 14 for animals treated only with VM and adapted for 6 and 9 days, respectively; and from the 15th to the 20th days for the other treatments. In this way, there are two analyzes in which the data were compared. Data were analyzed by PROC MIXED of SAS (2003), where residual normality (Shapiro–Wilk's and Kolmogorov–Smirnov's) and variance heterogeneity (GROUP option of SAS) tests were performed before the analysis of variance. The effect of the treatments was considered fixed; however, the effects of period and animal were considered random factors in the model. Response variables, such as the molar proportion of SCFA, NH_3_-N concentration, and protozoa counting were analyzed with repeated measures over time (33). The model included the same effects just described plus time and its interactions with treatments. Each variable analyzed as repeated measures was subjected to 8 covariance structures: unstructured, compound symmetric, heterogeneous compound symmetric, autoregressive of order one [AR (1)], heterogeneous first-order autoregressive [ARH (1)], Toeplitz, heterogeneous Toeplitz, and ante-dependence of order one [ANTE (1)]. The covariance structure that yielded the smaller Akaike and Schwarz's Bayesian criterion based on their −2 res log-likelihood was considered to provide the best fit.

For all response variables analyzed, the following contrasts were tested: (1) linear relationship between days of adaptation when only VM was fed (6, 9, and 14 d) and the dependent variable; (2) quadratic relationship between days of adaptation when only VM was fed (6, 9, and 14 d) and the dependent variable; (3) MONVM14 vs. VM14, and (4) MON14 vs. VM14. As days of adaptation were unequally spaced, we used a SAS macro (ORPOLY), which finds contrast coefficients for orthogonal polynomials for testing a quantitative factor variable and constructs CONTRAST statements using these values. Differences were considered significant at *P* < 0.05.

## 3. Results

### 3.1. Dry matter intake and ruminal pH

The results of DMI and ruminal pH on the 16th day of the experimental period are presented in Table 2. When VM was fed as the sole feed additive, no effect was observed (*P* > 0.69) on DMI; however, steers fed either MON14 or MONVM14 decreased DMI (*P* ≤ 0.04) in kg and as % of BW when compared to those fed VM14.

**Table 2:** Dry matter intake and ruminal pH of rumen cannulated Nellore cattle fed high concentrate diets containing sodium monensin (MON), virginiamycin (VM) or both on day 16 of the experimental period (or both during finishing periods).

**Item**	**Treatments** ^**a**^						***P-value***			
	**MON**	**MONVM**	**VM**				**MONVM14**	**MON14**	**VM effect** ^e^	
	**14**	**14**	**6**	**9**	**14**	**SEM** ^d^	**vs. VM14**	**vs. VM14**	**L**	**Q**
**DMI** ^b^										
Kg	8.99	8.88	9.37	9.76	9.62	0.4	0.01	0.04	0.78	0.69
% of BW^c^	2.18	2.16	2.33	2.35	2.33	0.74	0.02	0.03	0.94	0.73
**pH measurement**										
Mean pH	5.83	5.91	6.12	6.29	5.96	0.08	0.57	0.20	0.16	0.01
Maximum pH	6.83	6.75	6.90	7.04	7.03	0.10	0.03	0.11	0.33	0.51
Minimum pH	4.96	5.06	5.15	5.18	5.15	0.12	0.48	0.15	0.97	0.76
Duration pH < 5.2, h	1.14	2.30	0.77	0.82	1.59	0.77	0.50	0.27	0.15	0.43
Duration pH < 5.6, h	8.40	5.79	2.51	2.38	5.53	1.47	0.89	0.16	0.05	0.27
Duration pH < 6.2 h	18.62	16.72	10.11	8.14	15.34	1.64	0.64	0.35	0.05	0.01
Area < 5.2 pH × h	0.15	0.40	0.08	0.11	0.19	0.12	0.23	0.15	0.05	0.39
Area < 5.6 pH × h	1.87	1.98	0.71	0.70	1.60	0.58	0.62	0.88	0.05	0.32
Area < 6.2 pH × h	10.01	8.49	4.13	3.52	7.65	1.51	0.64	0.40	0.02	0.05
Temperature	39.07	39.20	39.40	39.12	39.24	0.07	0.71	0.02	0.03	0.04
Ox-redox potential	−372.09	−355.67	−376.33	−385.3	−382.9	12.76	0.05	0.51	0.70	0.69

^a^MON14: 27 mg of MON/kg of DM and adaption for 14 d; MON+VM14: 27 mg of MON/kg of DM and 25 mg of VM/kg of DM and adaption for 14 d; VM14: 25 mg of VM/kg of DM and adaption for 14 d; VM9: 25 mg of VM/kg of DM and adaption for 9 d; VM6: 25 mg of VM/kg of DM and adaption for 6 d;

^b^Dry matter intake;

^c^Body weight;

^d^Standard Error of Mean;

^e^L: linear and Q: quadratic responses for the effect of adaptation length in cattle fed only VM.

For rumen pH variables, no effects of treatments were observed (*P* > 0.15) for minimum pH and time below pH 5.2. However, time below pH < 5.6, time below pH < 6.2, area under pH 5.2, area under pH 5.6, and area under pH 6.2 decreased linearly (*P* ≤ 0.05; Table 2) by shortening the adaptation period from 14 to 6 d for cattle fed VM as a sole feed additive. Furthermore, mean rumen pH and temperature were affected quadratically (*P* ≤ 0.04) when the adaptation period was shortened from 14 to 6 d for cattle fed only VM as a feed additive. No differences (*P* > 0.15) in mean and minimum pH, as well as in time below pH 5.2, 5.6, and 6.2, and area under pH 5.2, 5.6, and 6.2 were observed when cattle fed VM14 was compared to steers consuming either MON14 or MONVM14. Besides that, cattle fed VM14 had greater maximum rumen pH (*P* = 0.03), and lower ox-redox potential (*P* = 0.02), than animals fed MONVM14. In addition, steers receiving VM14 slightly increased rumen temperature when compared to those fed MON14.

The results of DMI and ruminal pH on the 2nd day after adaptation period are presented in Supplementary Table A. As adaptation length was increased for animals fed VM, intake in kg and% BW increased linearly (*P* < 0.01; 8.37, 9.31 and 9.77 kg; 2.02, 2.21, 2.32 %BW for VM6, VM9 or VM14, respectively), with no differences for MON14 when compared to VM14 (9.17 kg and 2.29 %BW, respectively; *P* > 0.13). Furthermore, on day 2 after adaptation, as the adaptation time increased for animals fed only VM, the mean pH variable was quadratically affected (*P* = 0.03), and the highest value was observed for animals adapted at 9 days (5.94, 6.16 and 5.96 for VM6, VM9 or VM14, respectively). Moreover, a quadratic effect was observed for time below pH < 5.2 h (*P* = 0.01) and pH < 6.2 h (*P* = 0.01), and the lowest value was observed for animals adapted at 9 days for both pH < 5.2h (1.11, 0.72, and 1.59 for VM6, VM9 or VM14, respectively) and pH < 6.2 h (5.44, 3.11 and 5.53 for VM6, VM9, or VM14, respectively).

### 3.2. Feeding behavior and particle sorting

The results of feeding behavior and particle sorting on the 16th day of the experimental period are presented in Table 3. When cattle receiving VM14 were compared to animals fed MONVM14 and MON14, a decrease in RRDM and RRNDF was observed (*P* ≤ 0.05). Moreover, cattle consuming diets containing MON14 or VMMON14 had lesser DMI (*P* ≤ 0.02) than that fed VM14. Cattle fed MONVM14 also decreased (*P* < 0.01) NDF intake when compared to animals receiving VM14. It was observed that cattle receiving only VM as a feed additive increased RRDM and RRNDF quadratically (*P* ≤ 0.05) when the adaptation period was shortened from 14 to 6 d. Furthermore, NDF intake was linearly decreased (*P* = 0.05) by shortening the adaptation period from 14 to 6 d. No other differences (*P* > 0.08) were detected in terms of feeding behavior by shortening the adaptation period for cattle-fed VM as the sole feed additive. Regarding particle sorting, no differences were detected (*P* > 0.15) among cattle adapted for 14 days regardless of the feed additive fed. On the other hand, the sorting of long, medium, and fine particles was impacted quadratically by shortening the adaptation period from 14 to 6 days in cattle fed only VM.

**Table 3:** Feeding behavior and feed selectivity of rumen cannulated Nellore cattle fed high concentrate diets containing sodium monensin (MON), virginiamycin (VM) or both on the 16th day of the experimental period.

**Item**	**Treatments** ^**a**^						***P-value***			
	**MON**	**MONVM**	**VM**			**SEM** ^h^	**MONVM14**	**MON14vs**.	**VM effect** ^i^	
	**14**	**14**	**6**	**9**	**14**		**vs. VM14**	**VM14**	**L**	**Q**
**Feeding behavior**										
Time spent resting, min	847	875	832.43	785	851	35.02	0.56	0.92	0.68	0.14
Time spent ruminating, min	417	387	433.5	455	392	23.93	0.87	0.42	0.21	0.13
Time spent eating, min	169	166	163.66	186	184	18.27	0.22	0.3	0.2	0.34
Meal length, min	15.78	18.45	17.63	18.98	17.28	2.59	0.5	0.39	0.85	0.33
DMI^b^, Kg	9.88	9.72	10.92	10.36	11.08	0.46	0.01	0.02	0.74	0.13
DMI per meal, Kg	1.02	1.18	1.23	1.19	1.06	0.24	0.37	0.79	0.26	0.68
ERDM^c^, min/kg de DM	17.99	17.25	14.81	18.22	16.61	2.26	0.64	0.32	0.24	0.05
RRDM^d^, min/kg de DM	43.22	40.41	39.11	43.95	35.37	3.11	0.05	0.02	0.25	0.02
NDF^e^ intake	4.09	3.69	4.15	4.25	4.51	0.2	< 0.01	0.07	0.05	0.69
ERNDF^f^, min/kg de DM	43.91	45.84	38.82	45.02	40.96	6.23	0.29	0.52	0.67	0.22
RRNDF^g^, min/kg de DM	104.39	106.41	103.25	107.73	87.17	7.69	0.03	0.05	0.08	0.05
**Particle sorting**										
Long	0.91	0.96	0.87	1.02	0.87	0.1	0.46	0.75	0.98	0.05
Medium	1.07	1.05	1.08	1.16	1.02	0.05	0.66	0.45	0.47	0.04
Short	1.03	1.06	1.03	1.07	1.02	0.02	0.15	0.79	0.68	0.11
Fine	0.96	0.95	0.96	0.9	0.96	0.02	0.66	0.92	0.99	0.02

^a^MON14: 27 mg of MON/kg of DM and adaption for 14 d; MON+VM14: 27 mg of MON/kg of DM and 25 mg of VM/kg of DM and adaption for 14 d; VM14: 25 mg of VM/kg of DM and adaption for 14 d; VM9: 25 mg of VM/kg of DM and adaption for 9 d; VM6: 25 mg of VM/kg of DM and adaption for 6 d;

^b^Dry matter intake;

^c^Eating rate of dry matter;

^d^Rumination rate of dry matter;

^e^Neutral detergent fiber;

^f^Eating rate of NDF;

^g^Rumination rate of NDF;

^h^Standard Error of Mean;

^i^L: linear and Q: quadratic responses for the effect of adaptation length in cattle fed only VM.

The results of feeding behavior and particle sorting on the 2nd day after adaptation period are presented in Supplementary Table B. The RRDM was linearly decreased (*P* = 0.02) by shortening the adaptation period from 14 to 6 d (42.23, 41.29 and 35.37 for VM6, VM9 or VM14, respectively). Furthermore, time spent ruminating was affected quadratically (*P* = 0.05) when the adaptation period was shortened from 14 to 6 d for cattle fed only VM as a feed additive (428.00, 452.00 and 392.00 min for VM6, VM9 or VM14, respectively). Similarly, time spent eating was linearly decreased (*P* = 0.03) by shortening the adaptation period from 14 to 6 d (228.00, 214.00 and 184.00 min for VM6, VM9 or VM14, respectively).

### 3.3. Ruminal fermentation and protozoa counting

The results of ruminal fermentation end-products and total and differential counting of protozoa on the 16th day of the experimental period are presented in Table 4. Cattle receiving MON14 showed (*P* = 0.04) a greater concentration of propionate, but a lower (*P* = 0.01) concentration of butyrate when compared to animals fed VM14. Furthermore, the acetate:propionate ratio decreased (*P* ≤ 0.05) for cattle consuming MON14 and MONVM14 when compared to that fed VM14. It's noteworthy to mention that no differences in concentration of Acetate (*P* > 0.74), Lactate (*P* > 0.53), and N-NH_3_ (*P* > 0.18) were observed among animals adapted for 14 days. Regarding ruminal protozoa, Nellore cattle receiving MON14 and MONVM14 had greater populations of protozoa from the genus *Diplodinium* (*P* < 0.01) when compared to those from the control group fed VM14; however, no differences were noted in populations of protozoa from the genus *Dasytricha* (*P* > 0.15) in this study.

**Table 4:** Evaluation of ruminal fermentation products and differential protozoan counting of rumen cannulated Nellore cattle fed high-concentrate diets containing sodium monensin (MON), virginiamycin (VM), or both on the 16th day of the experimental period.

**Item**	**Treatments** ^**a**^						***P*** **-value**			
	**MON**	**MONVM**	**VM**				**MONVM14**	**MON14**	**VM effect** ^c^	
	**14**	**14**	**6**	**9**	**14**	**SEM** ^b^	**vs. VM14**	**vs. VM14**	**L**	**Q**
Acetate, mol/ 100 mol	60.03	58.75	65.04	61.68	59.98	2.13	0.74	0.99	0.17	0.78
Propionate, mol/ 100 mol	30.77	27.32	25.98	22.96	24.28	2.31	0.33	0.04	0.61	0.43
Butyrate, mol/ 100 mol	11.82	14.79	14.79	14.02	15.09	0.9	0.92	0.01	0.74	0.38
Total SCFA, mM^*^	102.62	100.86	106.16	98.66	99.34	4.21	0.81	0.6	0.31	0.46
Acet.: Prop.	2.14	2.24	2.6	2.8	2.61	0.17	0.05	0.04	0.99	0.45
Lactate mM	0.05	0.06	0.05	0.05	0.07	0.06	0.85	0.53	0.56	0.54
N-NH_3_ mg/ dl	7.03	8	7.99	6.07	7.11	0.62	0.18	0.99	0.26	0.04
Dasytricha ^×^ 10^c^/ ml	0.67	0.82	0.63	0.53	0.38	0.2	0.15	0.34	0.25	0.9
Isotricha × 10^c^/ ml^*^	1.63	1.2	2.74	2.88	1.3	0.29	0.78	0.48	< 0.01	0.02
Entodinium × 10^c^/ ml^*^	216.19	214.75	297.66	296.11	214.46	4.87	0.96	0.82	< 0.01	0.01
Diplodinium × 10^c^/ ml	29.28	27.07	23.29	26.83	16.99	0.92	< 0.01	< 0.01	< 0.01	< 0.01
Total × 10^c^ / ml^*^	247.78	243.84	325.09	326.35	233.14	5.64	0.23	0.11	< 0.01	< 0.01

^a^MON14: 27 mg of MON/kg of DM and adaption for 14 d; MON+VM14: 27 mg of MON/kg of DM and 25 mg of VM/kg of DM and adaption for 14 d; VM14: 25 mg of VM/kg of DM and adaption for 14 d; VM9: 25 mg of VM/kg of DM and adaption for 9 d; VM6: 25 mg of VM/kg of DM and adaption for 6 d;

^*^Treatment vs. collection time;

^b^Standard Error of Mean;

^c^L: linear and Q: quadratic responses for the effect of adaptation length in cattle fed only VM.

**Figure 2:**
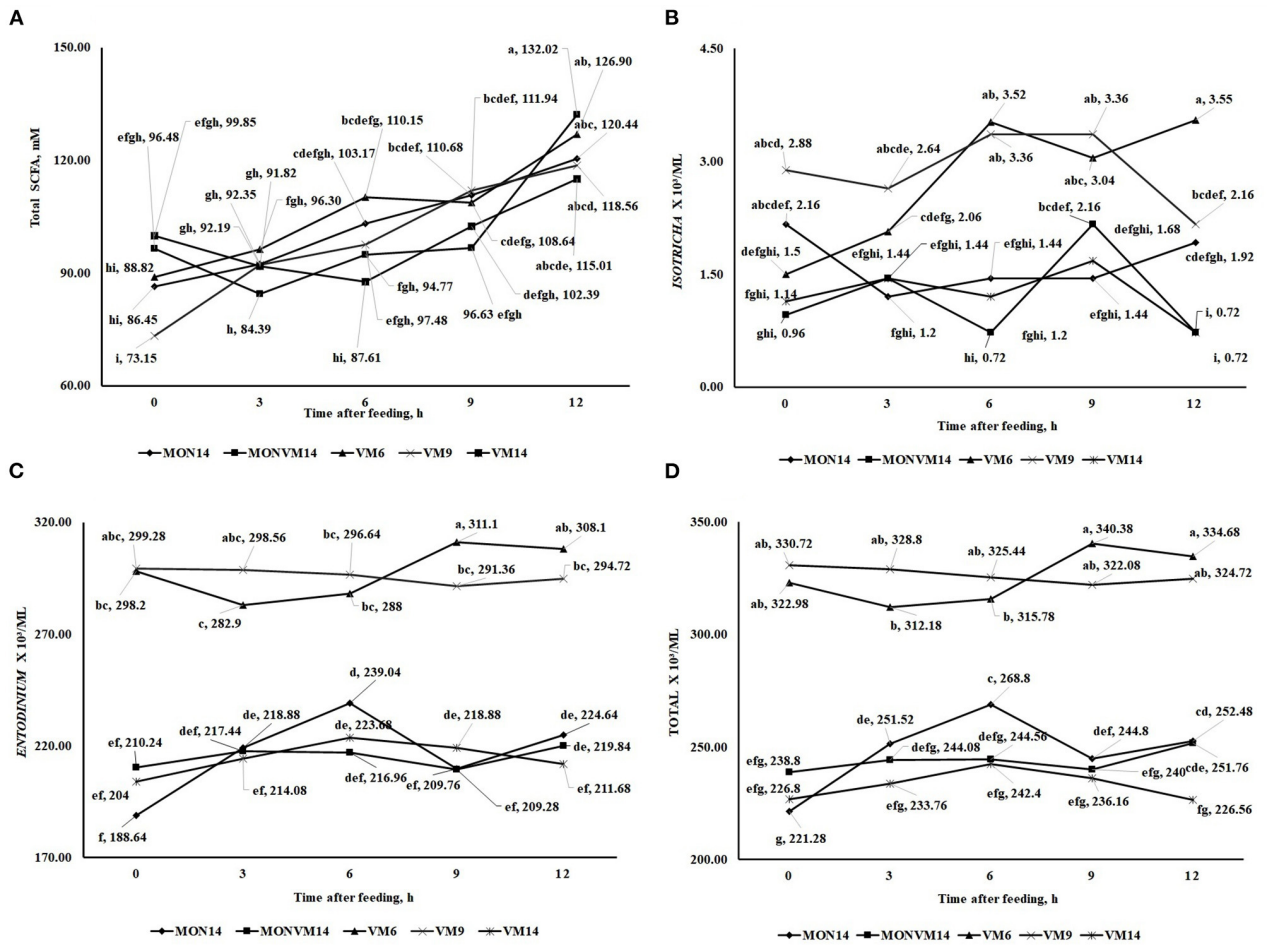
This is a figure with sub figures. **(A)** Interaction between treatments and hours after the treatment^1^ on the concentration of total (SCFA) fatty acid chain (mM) of rumen-cannulated Nellore cattle fed high-concentrate diets containing sodium monensin (MON), virginiamycin (VM) or both on the 16th day of the experimental period. **(B)** Interaction between treatments and hours after treatment on total ruminal Isotricha count (ml3/ml) of rumen-cannulated Nellore cattle fed high-concentrate diets containing sodium monensin (MON), virginiamycin (VM) or both on the 16th day of the experimental period. **(C)** Interaction between treatments and hours after treatment on total ruminal Entodinium count (ml3/ml) of rumen-cannulated Nellore cattle fed high-concentrate diets containing sodium monensin (MON), virginiamycin (VM) or both on the 16th day of the experimental period. **(D)** Interaction between treatments and hours after treatment on total ruminal protozoan count (ml3/ml) of rumen-cannulated Nellore cattle fed high-concentrate diets containing sodium monensin (MON), virginiamycin (VM) or both on the 16th day of the experimental period.

There was no effect of shortening the adaptation period from 14 to 6 days for Nellore cattle consuming only VM as feed additive (*P* > 0.10) for most variables evaluated, except for N-NH_3_ (*P* = 0.04) and populations of protozoa from the genus *Diplodinium* (*P* < 0.01) that presented a quadratic response. Moreover, it was observed an interaction between treatments and time for Total SCFA (*P* < 0.01), and populations of protozoa from the genus, *Isotricha* (*P* < 0.01), *Entodinium* (*P* < 0.01), and total protozoa (*P* < 0.01), which are shown in Figure 1. For total SCFA concentration, at hours 0 and 6 cattle receiving VM9 decreased the total concentration of SCFA (Figure 2A). For the variables of ciliated protozoa, cattle in VM6 and VM9 groups presented greater populations of *Isotricha* (Figure 2B), *Entodinium* (Figure 2C), and total protozoa (Figure 2D).

**Figure 3:**
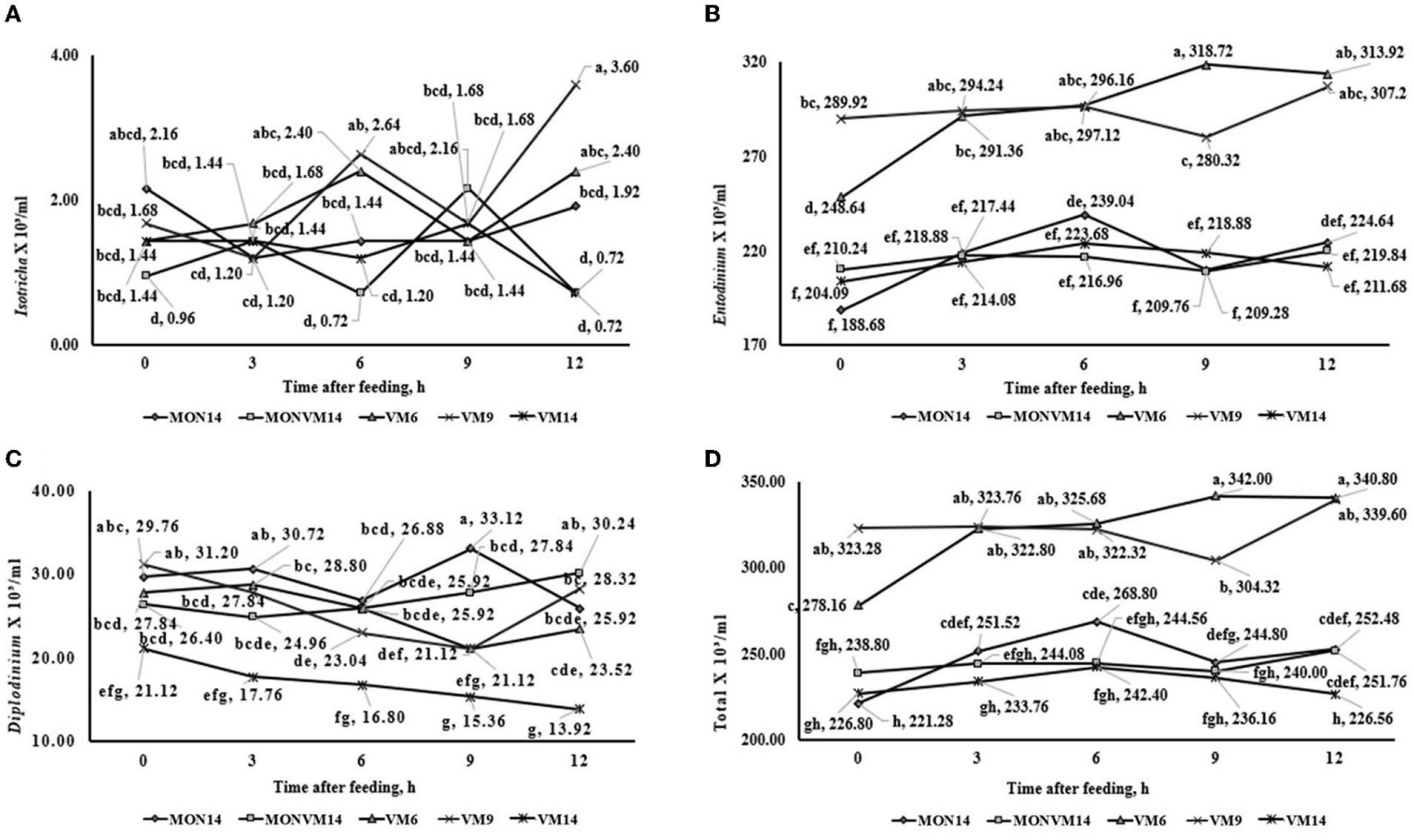
This is a figure with sub figures. **(A)** Interaction between treatments^2^ and hours after treatment on total ruminal Isotricha count (ml3/ml) of rumen-cannulated Nellore cattle fed high-concentrate diets containing sodium monensin (MON), virginiamycin (VM) or both on the 2th day after adaptation period. **(B)** Interaction between treatments and hours after treatment on total ruminal Entodinium count (ml3/ml) of rumen-cannulated Nellore cattle fed high-concentrate diets containing sodium monensin (MON), virginiamycin (VM) or both on the 2th day after adaptation period. **(C)** Interaction between treatments and hours after treatment on total ruminal Diplodinium count (ml3/ml) of rumen-cannulated Nellore cattle fed high-concentrate diets containing sodium monensin (MON), virginiamycin (VM) or both on the 2th day after adaptation period. **(D)** Interaction between treatments and hours after treatment on total ruminal protozoan count (ml3/ml) of rumen-cannulated Nellore cattle fed high-concentrate diets containing sodium monensin (MON), virginiamycin (VM) or both on the 2th day after adaptation period.

The results of ruminal fermentation end-products and total and differential counting of protozoa on the 2nd day after adaptation period are presented in Supplementary Table C. No effects of treatments were observed (*P* > 0.10) in concentration of Acetate, Lactate and total SCFA. Furthermore, N-NH3 concentration was affected quadratically (*P* = 0.05) when the adaptation period was shortened from 14 to 6 d for cattle fed only VM as a feed additive (6.72, 5.67 and 7.11 mg/ dl for VM6, VM9 or VM14, respectively). Regarding ruminal protozoa, it was observed an interaction between treatments and time for populations of protozoa from the genus *Isotricha* (*P* < 0.01), *Entodinium* (*P* < 0.01), *Diplodinium* (*P* < 0.01), and total protozoa (*P* < 0.01), which are shown in Figure 3. For the variables of ciliated protozoa, cattle in VM6 and VM9 groups presented greater populations of *Isotricha* (Figure 3A), *Entodinium* (Figure 3B), and total protozoa (Figure 3D). However, cattle fed VM14 presented smaller populations of *Diplodinium* (Figure 3C).

### 3.4. *In situ* degradability and total tract apparent digestibility

The results of *in situ* ruminal degradability of nutrients on the 16th day of the experimental period are presented in Table 5. Cattle fed MON14 decreased (*P* ≤ 0.05) ruminal degradability of DM, NDF, ADF, starch, NFE, and TDN, but increased CP degradability, when compared to animals consuming VM14. Likewise, cattle receiving MONVM14 had lower (*P* ≤ 0.04) ruminal degradability of DM, NDF, ADF, EE, starch, NFE, and TDN than those fed VM14. Furthermore, as the adaptation length was shortened for cattle consuming only VM as a feed additive, the degradability of DM, NDF, ADF, EE, and TDN decreased linearly (*P* ≤ 0.05) for animals that consumed only VM. In addition, it was observed (*P* = 0.05) a quadratic response for CP degradability when the adaptation length was shortened, in which cattle fed VM9 presented greater CP degradability.

**Table 5:** *In situ* degradability and total apparent digestibility for rumen cannulated cattle fed high concentrate diets containing sodium monensin (MON), virginiamycin (VM) or both on the 16th day of the experimental period.

**Item**	**Treatments** ^**a**^						***P-*** **value**			
	**MON**	**MONVM**	**VM**				**MONVM14**	**MON14**	**VM effects** ^e^	
	**14**	**14**	**6**	**9**	**14**	**SEM** ^d^	**vs. VM14**	**vs. VM14**	**L**	**Q**
***In situ*** **degradability**										
Dry Matter, %	69.90	63.39	71.46	73.81	74.55	0.60	< 0.01	< 0.01	0.01	0.53
Neutral Detergent Fiber, %	36.59	33.70	35.40	44.03	45.11	2.89	0.01	0.04	0.03	0.29
Acid Detergent Fiber^d^, %	25.93	23.60	20.06	28.36	32.70	3.05	0.04	0.05	0.01	0.58
Ethereal extract	84.88	71.90	78.02	81.05	84.32	3.16	0.04	0.89	0.04	0.95
Crude protein, %	75.35	61.78	62.26	71.55	66.20	2.90	0.28	0.03	0.36	0.05
Starch, %	86.50	80.51	93.89	93.23	95.51	1.26	< 0.01	< 0.01	0.35	0.31
NFE^b^, %	82.76	78.64	90.24	90.24	93.11	1.57	< 0.01	< 0.01	0.77	0.17
TDN^c^, %	69.74	63.10	71.01	72.65	73.02	0.68	< 0.01	< 0.01	0.05	0.43
**Total apparent digestibility**										
Dry Matter, %	75.10	74.16	74.15	71.97	75.45	0.29	< 0.01	0.30	< 0.01	< 0.01
Neutral Detergent Fiber, %	58.93	63.44	62.22	61.57	60.56	0.58	< 0.01	0.05	0.05	0.79
Acid Detergent Fiber, %	49.04	53.60	48.28	54.79	51.33	0.81	0.04	0.04	0.01	< 0.01
Ethereal extract	74.63	74.79	79.51	70.22	79.47	0.48	< 0.01	< 0.01	0.93	< 0.01
Crude protein, %	77.63	81.81	78.25	77.87	78.44	0.28	< 0.01	0.04	0.63	0.15
Starch, %	92.45	92.23	86.13	92.74	92.70	0.20	0.09	0.35	< 0.01	< 0.01
NFE, %	86.77	80.20	81.88	80.24	84.72	0.47	< 0.01	< 0.01	< 0.01	< 0.01
TDN, %	77.01	75.91	75.87	73.80	76.95	0.22	< 0.01	0.79	< 0.01	< 0.01

^a^MON14: 27 mg of MON/kg of DM and adaption for 14 d; MON+VM14: 27 mg of MON/kg of DM and 25 mg of VM/kg of DM and adaption for 14 d; VM14: 25 mg of VM/kg of DM and adaption for 14 d; VM9: 25 mg of VM/kg of DM and adaption for 9 d; VM6: 25 mg of VM/kg of DM and adaption for 6 d; ^b^Nitrogen free extract; ^c^Total digestible nutrients; ^d^Standard Error of Mean; 5L: linear and Q: quadratic responses for the effect of adaptation length in cattle fed only VM.

Concerning the total tract apparent digestibility, the results are in Table 5. Animals receiving MON14 decreased (*P* ≤ 0.05) digestibility of NDF, ADF, EE, and CP, but increased (*P* < 0.01) NFE digestibility when compared to cattle consuming VM14. Moreover, cattle receiving MONVM14 had lower (*P* ≤ 0.01) digestibility of DM, EE, NFE, and TDN, but greater (*P* ≤ 0.04) digestibility of NDF, ADF, and CP, when compared to animals fed VM14. When the adaptation period was shortened, the digestibility of DM, ADF, and EE responded quadratically (*P* < 0.01), in which cattle fed VM9 presented lower digestibility of DM and EE, and greater digestibility of ADF. Moreover, the digestibility of starch, NFE, and TDN decreased linearly (*P* < 0.01), but NDF digestibility increased linearly (*P* = 0.05), as the adaptation length was shortened.

The results of *in situ* ruminal degradability of nutrients on the 2nd day after adaptation period are presented in Supplementary Table D. Ruminal degradability of DM, NDF, ADF, and EE was affected linearly (*P* < 0.01) when the adaptation period was increased from 6 to 14 d for cattle fed only VM as a feed additive. Furthermore, when the adaptation period was shortened, the total apparent digestibility of DM, NDF, ADF, EE, CP, Starch, NFE and TDN responded quadratically (*P* < 0.01), in which cattle fed VM9 presented lower digestibility for all variables.

### 3.5. Ruminal dynamics

The results of ruminal dynamics on the 16th day of the experimental period are presented in Table 6. Cattle fed MON14 increased (*P* = 0.05) total mass in the rumen, expressed as % of BW, as well as lower (*P* = 0.03) Kt, expressed as %/h, when compared to animals receiving VM14. Likewise, animals fed MONVM14 showed a lower (*P* = 0.03) Kt, expressed as %/h than that fed VM14. In addition, cattle consuming MONVM14 presented greater (*P* = 0.05) ruminal DM content than cattle fed VM14. Regarding adaptation length, no effect was observed (*P* > 0.10) on ruminal dynamics when the adaptation period was shortened to either 9 or 6 days.

**Table 6:** Ruminal dynamics of rumen cannulated cattle fed high concentrate diets containing sodium monensin (MON), virginiamycin (VM), or both on the 16th day of the experimental period.

**Item**	**Treatments** ^**a**^						***P-value***			
	**MON**	**MONVM**	**VM**				**MONVM14**	**MON**	**VM effect** ^e^	
	**14**	**14**	**6**	**9**	**14**	**SEM** ^d^	**vs. VM14**	**vs. VM 14**	**L**	**Q**
Body weight, kg	413.40	412.59	414.55	419.89	412.62	21.21	0.99	0.85	0.67	0.11
Total liquid mass, Kg	33.23	32.42	33.24	32.64	31.94	1.60	0.67	0.27	0.30	0.96
Total solid mass, Kg	5.94	5.99	5.91	5.58	5.42	0.36	0.11	0.15	0.20	0.79
Total mass, Kg	39.17	38.42	39.14	38.23	37.36	1.91	0.45	0.20	0.24	0.98
Total liquid mass, % BW^b^	8.13	7.87	8.07	7.93	7.73	0.50	0.58	0.11	0.21	0.88
Total solid mass, % BW	1.44	1.46	1.43	1.36	1.32	0.11	0.11	0.14	0.23	0.90
Total mass, % BW	9.57	9.32	9.50	9.30	9.05	0.60	0.37	0.05	0.18	0.93
DM^c^ disappearance rate, Kg/h	0.39	0.39	0.44	0.45	0.43	0.67	0.22	0.18	0.88	0.41
Solid disappearance rate, %/ h	6.62	6.61	7.81	8.30	8.04	0.03	0.03	0.03	0.72	0.48
DM of rumen content, %	15.13	15.68	15.13	14.57	14.49	0.44	0.05	0.21	0.49	0.69

^a^MON14: 27 mg of MON/kg of DM and adaption for 14 d; MON+VM14: 27 mg of MON/kg of DM and 25 mg of VM/kg of DM and adaption for 14 d; VM14: 25 mg of VM/kg of DM and adaption for 14 d; VM9: 25 mg of VM/kg of DM and adaption for 9 d; VM6: 25 mg of VM/kg of DM and adaption for 6 d; ^b^Body weight, ^c^Dry matter; ^d^Standard Error of Mean; ^e^L: linear and Q: quadratic responses for the effect of adaptation length in cattle fed only VM.

The results of ruminal dynamics on the 2nd day after adaptation period are presented in Supplementary Table E. There was no linear or quadratic effect of shortening the adaptation period from 14 to 6 days for Nellore cattle fed only VM as feed additive (*P* > 0.09) for the variables evaluated on ruminal dynamics.

## 4. Discussion

The first part of this discussion section was to verify the effectiveness of VM in promoting a safe adaptation for cattle adapted to high-concentrate diets in 14 days when compared to MON and MON + VM. Based on the fact that the feeding of VM was as effective as MON and MON + VM to assure a good adaptation for Nellore cattle, the second part of the discussion was to evaluate the potential of shortening the adaptation period from 14 to 6 days when VM was added into high-concentrate diets as the sole feed additive.

### 4.1. Adaptation in 14 days

Considering only 14 days of adaptation, the DMI was higher for cattle that received only VM compared to animals that consumed diets containing MONVM or MON, and this result is clearly explained by the fact that VM does not decrease DMI (13). In contrast, the addition of MON in feedlot diets, associated or not with VM, leads to a reduction in DMI. This reduction in intake may be due to the longer ruminal retention time of dry matter, and to the increased production of propionic acid, which in ruminants is responsible for the regulation of animal satiety (14). These data differ from the reported by Salinas-Chavira (34), where Dutch steers consuming 88% concentrate containing different levels of VM (0, 16, 22.5 ppm) presented similar DMI when compared to cattle fed MON (28 ppm). Furthermore, the positive effect on DMI promoted by feeding only VM had no impact on ruminal pH, which emphasizes the efficiency of VM in controlling rumen fermentation. The replacement of MON and MONVM by only VM as the sole feed additive in the diet increased NDF intake, and also the RRDM and RRNDF, which may have helped cattle receiving VM to cope with ruminal acidification at greater DMI.

On the other hand, cattle consuming only MON had higher concentrations of propionic acid, which is in agreement with Duffield et al. (12). In a companion study, considering 14 days of adaptation, Rigueiro et al. (35) reported that cattle fed only MON improved feed efficiency when compared to those fed only VM, showing that ionophores, such as MON, increase energy availability when added to high-energy diets. According to Lanna and Medeiros (36), the increase in propionate concentration in the rumen is associated with the reduction of DMI, a phenomenon known by the chemostatic mechanism. In addition, cattle receiving either MON or VM did not differ in terms of total SCFA concentrations overall; however, these feed additives tested in this study slightly shift concentrations of specific SCFA, such as the reduced acetate:propionate ratio presented by cattle fed MON. The rumen pH and N-NH3 concentration have a great impact on the main end products of rumen fermentation, which are the SCFA and the microbial protein, main sources of energy and amino acids for the animal. In this context, when SCFA accumulates and ruminal pH declines, urea cycling is compromised, resulting on a reduced urea infux and N-NH3 concentrations in the rumen (37, 38). In the present study, no effect of adaptation period was observed for SCFA concentrations in animals fed only VM. In addition, animals adapted for 14 days had higher N-NH3 concentrations, because the higher DMI observed for this treatment, resulting in more N-NH3 availability in the rumen; whereas for animals adapted for 6 days this higher N-NH3 concentration can be explained by not having enough microorganisms, due to the higher count of ciliated protozoa for this treatment, which use bacteria as a source of amino acids and nucleic acids (39).

Moreover, animals that consumed only VM had smaller numbers of *Diplodinium* genus in the rumen, which may be associated with an increased passage rate resulting from greater DMI presented by these animals, since there was no difference involving rumen pH variables of these animals. According to Frazolin and Dehority (40), diets containing high levels of energy may lead to a reduction in the protozoan population due to either reduced rumen pH or increased passage rate. It is noteworthy to mention that VM-fed cattle presented greater Kt, as well as increased ruminal degradability and total tract digestibility of most nutrients than those fed either MON or MONVM, which may lead to the inference that *Diplodinium* protozoa have been washed out of the rumen. Also, the feeding of VM decreased ruminal DM content, which is in agreement with the fact that the rumen passage rate was faster for these animals, but not enough to negatively impact the ruminal degradability of nutrients. These results corroborate the data from Poos et al. (41), who reported that in sheep diets with increasing levels of MON (0, 22, and 38 ppm), there was a reduction in ruminal degradability of DM and fiber. Ruminal degradation of CP may be also reduced with the inclusion of ionophores resulting in an increase in the amount of protein that bypasses the rumen (42). However, this study cannot confirm this.

When the adaptation period of 14 days was adopted, changes in ruminal fermentation patterns and in nutrient disappearance across the digestive tract were detected when MON and MONVM were replaced by VM as the sole feed additive in the diet; however, none of these changes negatively impacted the variables related to the adaptation period in this study. Thus, cattle fed VM can be safely adapted for 14 days to high-concentrate diets.

### 4.2. Adaptation in < 14 days

This section will discuss the effects of shortening the adaptation period from 14 to either 6 or 9 days using VM as the sole feed additive.

The shortening of the adaptation period did not affect the DMI, however, it changed the feeding behavior of Nellore that were adapted in < 14 days since they sorted for long and medium particles and against fine particles. Dado and Allen (31) reported that cattle may sort for longer diet particles and avoid diet fines to cope with ruminal acidification. Likewise, cattle adapted for either 9 or 6 days took longer to consume and ruminate a kg of DM, which is a sign of unproper ruminal function (43). This mechanism of animal defense against ruminal acidosis may have collaborated to decrease the pH duration below 5.6 and 6.2, resulting in a smaller area under pH 5.2, 5.6, and 6.2 for cattle adapted in < 14 days. As a result of increasing ruminal pH, ciliated protozoa numbers, such as *Isotricha, Entodinium*, and *Diplodinium* also increased, since they are sensitive to low pH (3), in cattle adapted in < 14 days.

The change observed in feeding behavior and particle sorting when cattle were adapted for either 6 or 9 days also negatively impacted ruminal degradability and total tract digestibility of most nutrients evaluated. Furthermore, this decrease in ruminal degradability of nutrients may have contributed to reducing ruminal fermentation, resulting in an increase in ruminal pH, and in the numbers of ciliated protozoa as well. Nagaraja and Tigtemeyer (3) reported that ruminal protozoa consume bacteria in order to control ruminal acidification to survive, and this may be one of the factors to explain the decrease in nutrient disappearance across the cattle's digestive tract. It's noteworthy to mention that the shortening of the adaptation did not negatively impact Kt, and the lower ruminal degradability of nutrients may have been offset by a faster passage rate since cattle adapted for either 6 sorted for long and medium particles. In a companion study, Rigueiro et al. (35) reported that shortening the adaptation period from 14 to either 9- or 6-days compromises carcass fat deposition, disrupts feeding behavior patterns, and does not promote any positive effect on animal performance and on development of both the rumen and cecum epithelium.

Therefore, despite maintaining adequate ruminal pH, the pH-related variables evaluated in this study only demonstrate that cattle were able to self-regulate to avoid acidosis. Moreover, it is noteworthy to mention that during the experimental period, an animal while on VM6 treatment presented intensive bloat when promoted to the finishing diet. The steer returned to the study in the next experimental period without presenting any signs of clinical bloat or acidosis for the rest of the study. The short time of 6 days to adapt to the finishing diet may have not allowed the animal self-regulate its behavior to avoid ruminal acidosis.

## 5. Conclusion

Nellore cattle adapted well to finishing diets, containing only VM as a feed additive, for a period of 14 days. However, it is not recommended to shorten the adaptation length of these animals to either 6 or 9 days without negatively impacting nutrient disappearance and ruminal fermentation patterns.

## Data availability statement

The raw data supporting the conclusions of this article will be made available by the authors, without undue reservation.

## Ethics statement

The animal study was reviewed and approved by the São Paulo State University Ethical Committee for Animal Research (protocol number 02/2017.R1- CEUA).

## Author contributions

MS and DM designed the experiment and wrote the manuscript. MS, AR, AS, CS, AA, ED, LF, LS, KS, VC, and BD conducted the experiment. MS performed the laboratory and data analyses. MS, JS, and DM provided intellectual input. All authors edited and approved the manuscript submission.

## Footnotes

^1^MON14, 27 mg of MON/kg of DM and adaption for 14 d; MON+VM148 27 mg of MON/kg of DM and 25 mg of VM/kg of DM and adaption for 14 d; VM14, 25 mg of VM/kg of DM and adaption for 14 d; VM9, 25 mg of VM/kg of DM and adaption for 9 d; VM6, 25 mg of VM/kg of DM and adaption for 6 d. ^a − i^Means without a common superscript letter differ (*P* < 0.01); pooled SEM (2.41, 0.29, 4.87, and 5.64).

^2^MON14, 27 mg of MON/kg of DM and adaption for 14 d; MON+VM14, 27 mg of MON/kg of DM and 25 mg of VM/kg of DM and adaption for 14 d; VM14, 25 mg of VM/kg of DM and adaption for 14 d; VM9, 25 mg of VM/kg of DM and adaption for 9 d; VM6, 25 mg of VM/kg of DM and adaption for 6 d. ^a − h^Means without a common superscript letter differ (P < 0.01); pooled SEM (0.28, 4.88, 0.97, and 5.06).
