# Supplementary material for: Shortening the adaptation of Nellore cattle to high-concentrate diets using only virginiamycin as sole feed additive negatively impacts ruminal fermentation and nutrient utilization
Source: Front Vet Sci. 2023 Mar 29;10:1089903. doi: 10.3389/fvets.2023.1089903 (PMC10090337; doi:10.3389/fvets.2023.1089903)
Supplement: Supplementary file 1 [file Data_Sheet_1.docx]

**Table A.** Dry matter intake and ruminal pH of rumen cannulated Nellore cattle fed high concentrate diets containing sodium monensin (MON), virginiamycin (VM) or both on day 2 after adaptation period.

| Item | Treatments^1^ | | | | |  | *P-value* | | | | |
| --- | --- | --- | --- | --- | --- | --- | --- | --- | --- | --- | --- |
|  | MON | MONVM | VM | | |  | MONVM14 vs. VM14 | MON14 vs. VM14 | VM effect^5^ | | |
|  | 14 | 14 | 6 | 9 | 14 | SEM^4^ |  |  | L | Q |  |
| DMI^2^ |  |  |  |  |  |  |  |  |  |  |  |
| Kg | 9.17 | 9.05 | 8.37 | 9.31 | 9.77 | 0.55 | 0.08 | 0.13 | <0.01 | 0.48 |  |
| % of BW^3^ | 2.29 | 2.06 | 2.02 | 2.21 | 2.32 | 0.09 | 0.01 | 0.72 | <0.01 | 0.61 |  |
| pH measurement | | | | | |  |  |  |  |  |  |
| Mean pH | 5.83 | 5.91 | 5.94 | 6.16 | 5.96 | 0.08 | 0.59 | 0.22 | 0.79 | 0.03 |  |
| Maximum pH | 6.83 | 6.75 | 6.67 | 6.92 | 7.03 | 0.10 | 0.05 | 0.14 | 0.01 | 0.55 |  |
| Minimum pH | 4.96 | 5.06 | 5.06 | 5.18 | 5.15 | 0.11 | 0.51 | 0.17 | 0.48 | 0.49 |  |
| Duration pH <5.2, h | 1.14 | 2.30 | 1.11 | 0.72 | 1.59 | 0.85 | 0.93 | 0.31 | 0.75 | 0.01 |  |
| Duration pH <5.6, h | 8.40 | 5.79 | 5.44 | 3.11 | 5.53 | 1.31 | 0.93 | 0.28 | 0.89 | 0.11 |  |
| Duration pH <6.2 h | 18.62 | 16.72 | 17.40 | 11.61 | 15.34 | 1.82 | 0.51 | 0.36 | 0.74 | 0.01 |  |
| Area < 5.2 pH x h | 0.15 | 0.40 | 0.19 | 0.11 | 0.19 | 0.34 | 0.33 | 0.24 | 0.34 | 0.31 |  |
| Area < 5.6 pH x h | 0.59 | 1.98 | 1.20 | 0.70 | 1.61 | 0.51 | 0.76 | 0.91 | 0.51 | 0.21 |  |
| Area < 6.2 pH x h | 1.52 | 8.49 | 6.59 | 4.76 | 7.65 | 0.52 | 0.80 | 0.58 | 0.52 | 0.17 |  |
| Temperature | 39.07 | 39.21 | 39.27 | 39.16 | 39.24 | 0.072 | 0.67 | 0.07 | 0.80 | 0.25 |  |
| Ox-redox potential | -372.09 | -355.67 | -388.24 | -388.76 | -382.9 | 9.26 | 0.04 | 0.39 | 0.67 | 0.77 |  |

^1^MON14: 27 mg of MON/kg of DM and adaption for 14 d; MON+VM14: 27 mg of MON/kg of DM and 25 mg of VM/kg of DM and adaption for 14 d; VM14: 25 mg of VM/kg of DM and adaption for 14 d; VM9: 25 mg of VM/kg of DM and adaption for 9 d; VM6: 25 mg of VM/kg of DM and adaption for 6 d; ^2^Dry matter intake; ^3^Body weight; ^4^Standard Error of Mean; ^5^L: linear and Q: quadratic responses for the effect of adaptation length in cattle fed only VM.

**Table B.** Feeding behavior and feed selectivity of rumen cannulated Nellore cattle fed high concentrate diets containing sodium monensin (MON), virginiamycin (VM) or both on day 2 after adaptation period.

| Item | | Treatments^1^ | | | | |  | *P-value* | | | |
| --- | --- | --- | --- | --- | --- | --- | --- | --- | --- | --- | --- |
|  |  | MON | MONVM | VM | | | SEM^8^ | MONVM14  vs. VM14 | MON14vs.  VM14 | VM effect^9^ | |
|  |  | 14 | 14 | 6 | 9 | 14 |  |  |  | L | Q |
| Feeding behavior | |  |  |  |  |  |  |  |  |  |  |
| Time spent resting, min | | 847.00 | 875.00 | 775.00 | 760.00 | 851.00 | 33.04 | 0.53 | 0.92 | 0.05 | 0.12 |
| Time spent ruminating, min | | 417.00 | 387.00 | 428.00 | 452.00 | 392.00 | 22.96 | 0.84 | 0.33 | 0.17 | 0.05 |
| Time spent eating, min | | 169.00 | 166.00 | 228.00 | 214.00 | 184.00 | 18.67 | 0.36 | 0.44 | 0.03 | 0.63 |
| Meal length, min | | 15.78 | 18.45 | 16.57 | 17.76 | 17.28 | 1.98 | 0.55 | 0.45 | 0.72 | 0.63 |
| DMI^2^, Kg | | 9.88 | 9.72 | 10.34 | 10.96 | 11.08 | 0.49 | 0.01 | 0.02 | 0.04 | 0.55 |
| DMI per meal, Kg | | 1.02 | 1.18 | 0.82 | 0.97 | 1.06 | 0.19 | 0.36 | 0.79 | 0.04 | 0.81 |
| ERDM^3^, min/kg de DM | | 17.99 | 17.25 | 22.60 | 19.59 | 16.61 | 2.29 | 0.73 | 0.45 | <0.01 | 0.99 |
| RRDM^4^, min/kg de DM | | 43.22 | 40.41 | 42.23 | 41.29 | 35.37 | 3.21 | 0.05 | 0.01 | 0.02 | 0.31 |
| NDF^5^ intake | | 4.09 | 3.69 | 3.92 | 4.44 | 4.51 | 0.28 | 0.01 | 0.18 | 0.05 | 0.38 |
| ERNDF^6^, min/kg de DM | | 43.91 | 45.84 | 61.85 | 51.70 | 40.96 | 7.64 | 0.49 | 0.67 | 0.01 | 0.96 |
| RRNDF^7^, min/kg de DM | | 104.39 | 106.41 | 115.00 | 106.03 | 87.17 | 10.49 | 0.05 | 0.05 | 0.02 | 0.58 |
| Particle sorting | |  |  |  |  |  |  |  |  |  |  |
| Long |  | 0.91 | 0.96 | 0.81 | 0.83 | 0.87 | 0.09 | 0.42 | 0.73 | 0.55 | 0.96 |
| Medium |  | 1.07 | 1.05 | 1.04 | 0.95 | 1.02 | 0.05 | 0.59 | 0.45 | 0.50 | 0.05 |
| Short |  | 1.03 | 1.06 | 1.04 | 1.03 | 1.02 | 0.02 | 0.11 | 0.77 | 0.44 | 0.81 |
| Fine |  | 0.96 | 0.95 | 0.96 | 0.93 | 0.96 | 0.02 | 0.64 | 0.91 | 0.95 | 0.29 |

^1^MON14: 27 mg of MON/kg of DM and adaption for 14 d; MON+VM14: 27 mg of MON/kg of DM and 25 mg of VM/kg of DM and adaption for 14 d; VM14: 25 mg of VM/kg of DM and adaption for 14 d; VM9: 25 mg of VM/kg of DM and adaption for 9 d; VM6: 25 mg of VM/kg of DM and adaption for 6 d; ^2^Dry matter intake; ^3^Eating rate of dry matter; ^4^Rumination rate of dry matter; ^5^Neutral detergent fiber; ^6^Eating rate of NDF; ^7^Rumination rate of NDF; ^8^Standard Error of Mean; ^9^L: linear and Q: quadratic responses for the effect of adaptation length in cattle fed only VM.

**Table C.** Evaluation of ruminal fermentation products and differential protozoan counting of rumen cannulated Nellore cattle fed high-concentrate diets containing sodium monensin (MON), virginiamycin (VM), or both on day 2 after adaptation period.

| Item | Treatments^1^ | | | | |  | *P-value* | | | |
| --- | --- | --- | --- | --- | --- | --- | --- | --- | --- | --- |
|  | MON | MONVM | VM | | |  | MONVM14 vs. VM14 | MON14 vs.  VM14 | VM effect^3^ | |
|  | 14 | 14 | 6 | 9 | 14 | SEM^2^ |  |  | L | Q |
| Acetate, mol/ 100 mol | 60.03 | 58.75 | 63.73 | 66.10 | 59.98 | 2.38 | 0.77 | 0.99 | 0.50 | 0.27 |
| Propionate, mol/ 100 mol | 30.77 | 27.32 | 22.58 | 27.70 | 24.28 | 2.72 | 0.38 | 0.04 | 0.62 | 0.16 |
| Butyrate, mol/ 100 mol | 11.82 | 14.79 | 15.87 | 15.19 | 15.09 | 1.18 | 0.92 | 0.02 | 0.70 | 0.86 |
| Total SCFA, mM^*^ | 102.62 | 100.86 | 102.17 | 108.98 | 99.34 | 4.88 | 0.83 | 0.64 | 0.68 | 0.18 |
| Acet.: Prop. | 2.14 | 2.24 | 3.03 | 2.57 | 2.61 | 0.19 | 0.05 | 0.04 | 0.34 | 0.33 |
| Lactate mM | 0.06 | 0.07 | 0.06 | 0.06 | 0.07 | 0.00 | 0.76 | 0.30 | 0.53 | 0.48 |
| N-NH_3_ mg/ dl | 7.03 | 8.00 | 6.72 | 5.67 | 7.11 | 0.45 | 0.33 | 0.87 | 0.52 | <0.01 |
| *Dasytricha* x 10^3^/ ml | 0.67 | 0.82 | 0.62 | 0.624 | 0.38 | 0.27 | 0.106 | 0.32 | 0.15 | 0.64 |
| *Isotricha* x 10^3^/ ml^*^ | 1.63 | 1.20 | 1.87 | 2.16 | 1.30 | 0.28 | 0.83 | 0.41 | 0.03 | 0.03 |
| *Entodinium* x 10^3^/ ml^*^ | 216.19 | 214.75 | 293.95 | 293.57 | 214.46 | 4.88 | 0.03 | 0.28 | <0.01 | 0.03 |
| *Diplodinium* x 10^3^/ ml | 29.28 | 27.07 | 25.44 | 26.30 | 16.99 | 0.97 | <0.01 | <0.01 | <0.01 | <0.01 |
| Total x 10^3^ / ml^*^ | 247.78 | 243.84 | 321.89 | 322.66 | 233.14 | 5.06 | 0.13 | 0.08 | <0.01 | <0.01 |

^1^MON14: 27 mg of MON/kg of DM and adaption for 14 d; MON+VM14: 27 mg of MON/kg of DM and 25 mg of VM/kg of DM and adaption for 14 d; VM14: 25 mg of VM/kg of DM and adaption for 14 d; VM9: 25 mg of VM/kg of DM and adaption for 9 d; VM6: 25 mg of VM/kg of DM and adaption for 6 d; ^*^Treatment vs. collection time; ^2^Standard Error of Mean; ^3^L: linear and Q: quadratic responses for the effect of adaptation length in cattle fed only VM.

**Table D.** In situ degradability and total apparent digestibility for rumen cannulated cattle fed high concentrate diets containing sodium monensin (MON), virginiamycin (VM) or both on day 2 after adaptation period.

| ITEM | | Treatments^1^ | | | | |  | *P-value* | | | |  |
| --- | --- | --- | --- | --- | --- | --- | --- | --- | --- | --- | --- | --- |
|  |  | MON | MONVM | VM | | |  | MONVM14 vs. VM14 | MON14 vs. VM14 | VM effects^5^ | |  |
|  |  | 14 | 14 | 6 | 9 | 14 | SEM^4^ |  |  | L | Q | |
| *In situ degradability* | |  |  |  |  |  |  |  |  |  |  | |
| Dry Matter, % | | 69.90 | 63.39 | 63.71 | 72.89 | 74.56 | 0.54 | <0.01 | <0.01 | <0.01 | <0.01 | |
| Neutral Detergent Fiber, % | | 36.58 | 33.70 | 28.22 | 37.03 | 45.11 | 2.58 | <0.01 | 0.03 | <0.01 | 0.90 | |
| Acid Detergent Fiber^4^, % | | 25.93 | 23.60 | 18.99 | 27.07 | 32.70 | 3.24 | 0.03 | 0.09 | <0.01 | 0.72 | |
| Ethereal extract | | 84.88 | 71.90 | 70.79 | 81.08 | 84.31 | 4.91 | 0.56 | 0.05 | 0.04 | 0.06 | |
| Crude protein, % | | 75.35 | 61.78 | 62.75 | 63.68 | 66.20 | 2.48 | 0.09 | <0.01 | 0.17 | 0.71 | |
| Starch, % | | 86.50 | 80.51 | 81.17 | 94.33 | 95.51 | 1.07 | <0.01 | <0.01 | <0.01 | <0.01 | |
| NFE^2^, % | | 82.76 | 78.64 | 81.97 | 95.08 | 93.11 | 1.78 | <0.01 | <0.01 | <0.01 | <0.01 | |
| TDN^4^, % | | 69.74 | 63.10 | 63.48 | 71.89 | 73.02 | 0.69 | <0.01 | <0.01 | <0.01 | <0.01 | |
| Total apparent digestibility | |  |  |  |  |  |  |  |  |  |  | |
| Dry Matter, % | | 75.10 | 74.16 | 72.85 | 63.50 | 75.45 | 0.43 | 0.04 | 0.56 | <0.01 | <0.01 | |
| Neutral Detergent Fiber, % |  | 58.93 | 63.44 | 54.47 | 50.13 | 60.56 | 1.42 | 0.12 | 0.37 | <0.01 | <0.01 | |
| Acid Detergent Fiber, % |  | 49.04 | 53.60 | 44.33 | 41.10 | 51.33 | 0.98 | 0.09 | 0.08 | <0.01 | <0.01 | |
| Ethereal extract | | 74.63 | 74.79 | 78.96 | 76.09 | 79.47 | 0.61 | <0.01 | <0.01 | 0.54 | <0.01 | |
| Crude protein, % | | 77.63 | 81.91 | 77.86 | 73.65 | 78.44 | 0.36 | <0.01 | 0.10 | 0.24 | <0.01 | |
| Starch, % | | 92.45 | 92.23 | 93.01 | 90.87 | 92.70 | 0.18 | 0.07 | 0.31 | 0.49 | <0.01 | |
| NFE, % | | 86.77 | 80.19 | 83.45 | 69.42 | 84.72 | 0.54 | <0.01 | <0.01 | 0.03 | <0.01 | |
| TDN, % | | 77.01 | 75.91 | 74.97 | 65.37 | 76.95 | 0.22 | <0.01 | 0.78 | <0.01 | <0.01 | |

^1^MON14: 27 mg of MON/kg of DM and adaption for 14 d; MON+VM14: 27 mg of MON/kg of DM and 25 mg of VM/kg of DM and adaption for 14 d; VM14: 25 mg of VM/kg of DM and adaption for 14 d; VM9: 25 mg of VM/kg of DM and adaption for 9 d; VM6: 25 mg of VM/kg of DM and adaption for 6 d; ^2^ Nitrogen free extract; ^3^Total digestible nutrients; ^4^Standard Error of Mean; ^5^L: linear and Q: quadratic responses for the effect of adaptation length in cattle fed only VM.

**Table E.** Ruminal dynamics of rumen cannulated cattle fed high concentrate diets containing sodium monensin (MON), virginiamycin (VM), or both on day 2 after adaptation period.

| Item | | Treatments^1^ | | | | |  | *P-value* | | | |
| --- | --- | --- | --- | --- | --- | --- | --- | --- | --- | --- | --- |
|  |  | MON | MONVM | VM | | |  | MONVM14 vs. VM14 | MON vs. VM 14 | VM effect^5^ | |
|  |  | 14 | 14 | 6 | 9 | 14 | SEM^4^ |  |  | L | Q |
| Body weight, kg | | 413.40 | 412.59 | 413.24 | 422.45 | 412.62 | 21.71 | 0.99 | 0.90 | 0.92 | 0.09 |
| Total liquid mass, Kg | | 33.23 | 33.42 | 33.15 | 33.47 | 31.94 | 1.77 | 0.72 | 0.35 | 0.38 | 0.44 |
| Total solid mass, Kg | | 5.94 | 5.99 | 5.56 | 5.66 | 5.42 | 0.35 | 0.10 | 0.13 | 0.68 | 0.57 |
| Total mass, Kg | | 39.17 | 38.42 | 38.71 | 39.13 | 37.36 | 2.06 | 0.50 | 0.26 | 0.40 | 0.43 |
| Total liquid mass, % BW^2^ | | 8.13 | 7.87 | 8.15 | 8.11 | 7.73 | 0.57 | 0.73 | 0.31 | 0.29 | 0.61 |
| Total solid mass, % BW | | 1.44 | 1.46 | 1.38 | 1.38 | 1.32 | 0.12 | 0.14 | 0.18 | 0.50 | 0.66 |
| Total mass, % BW | | 9.57 | 9.32 | 9.53 | 9.49 | 9.05 | 0.68 | 0.55 | 0.26 | 0.30 | 0.60 |
| DM^3^ disappearance rate, Kg/h |  | 0.39 | 0.39 | 0.47 | 0.44 | 0.43 | 0.03 | 0.28 | 0.24 | 0.24 | 0.67 |
| Solid disappearance rate, %/ h |  | 6.62 | 6.61 | 8.63 | 7.83 | 8.04 | 0.52 | 0.03 | 0.03 | 0.33 | 0.33 |
| DM of rumen content, % | | 15.13 | 15.68 | 14.36 | 14.44 | 14.49 | 0.42 | 0.05 | 0.28 | 0.83 | 0.97 |

^1^MON14: 27 mg of MON/kg of DM and adaption for 14 d; MON+VM14: 27 mg of MON/kg of DM and 25 mg of VM/kg of DM and adaption for 14 d; VM14: 25 mg of VM/kg of DM and adaption for 14 d; VM9: 25 mg of VM/kg of DM and adaption for 9 d; VM6: 25 mg of VM/kg of DM and adaption for 6 d; ^2^Body weight, ^3^Dry matter; ^5^Standard Error of Mean; ^6^L: linear and Q: quadratic responses for the effect of adaptation length in cattle fed only VM.
